# What can we learn from an intersectionality-informed description of study participants? Results from the German National Cohort

**DOI:** 10.1186/s12939-025-02521-3

**Published:** 2025-05-26

**Authors:** Philipp Jaehn, Stefan Rach, Gabriele Bolte, Rafael Mikolajczyk, Sibille Merz, Paula Sofia Herrera-Espejel, Tilman Brand, Amand Führer, Klaus Berger, Henning Teismann, Barbara Bohn, Lena Koch-Gallenkamp, Hermann Brenner, Carolina J. Klett-Tammen, Stefanie Castell, Nina Ebert, Carina Emmel, Börge Schmidt, Sylvia Gastell, Matthias B. Schulze, Nadia Obi, Volker Harth, Bernd Holleczek, Stefanie Jaskulski, Verena Katzke, Rudolf Kaaks, Stefan N. Willich, Thomas Keil, Andrea Weber, Michael Leitzmann, Kerstin Wirkner, Claudia Meinke-Franze, Sabine Schipf, Tamara Schikowski, Alexandra Schneider, S. Claire Slesinski, Ilais Moreno-Velásquez, Tobias Pischon, Christine Holmberg

**Affiliations:** 1 Institute of Social Medicine and Epidemiology, Brandenburg Medical School Theodor Fontane, Hochstraße 15, Brandenburg an der Havel, 14770 Germany; 2 Faculty of Health Sciences Brandenburg, Brandenburg Medical School Theodor Fontane, Potsdam, Germany; 3 Leibniz Institute for Prevention Research and Epidemiology - BIPS, Bremen, Germany; 4 Department of Social Epidemiology, Institute of Public Health and Nursing Research, University of Bremen, Bremen, Germany; 5 Health Sciences Bremen, University of Bremen, Bremen, Germany; 6 Institute for Medical Epidemiology, Biometrics and Informatics, Martin Luther University Halle-Wittenberg, Halle, Germany; 7 Institute of Epidemiology and Social Medicine, University of Münster, Münster, Germany; 8 NAKO e.V., Heidelberg, Germany; 9 Division of Clinical Epidemiology and Aging Research, German Cancer Research Center (DKFZ), Heidelberg, Germany; 10 German Cancer Consortium (DKTK), German Cancer Research Center (DKFZ), Heidelberg, Germany; 11 Department for Epidemiology, Helmholtz Centre for Infection Research, Brunswick, Germany; 12 Institute for Biometrics and Epidemiology, German Diabetes Center, Leibniz Center for Diabetes Research, Heinrich Heine University Düsseldorf, Düsseldorf, Germany; 13 Institute for Medical Informatics, Biometry and Epidemiology, University Hospital Essen, University of Duisburg-Essen, Essen, Germany; 14 NAKO Study Center, German Institute of Human Nutrition Potsdam-Rehbruecke, Potsdam, Germany; 15 Department of Molecular Epidemiology, German Institute of Human Nutrition Potsdam-Rehbruecke, Potsdam, Germany; 16 Institute of Nutritional Science, University of Potsdam, Nuthetal, Germany; 17 Institute for Occupational and Maritime Medicine (ZfAM), University Medical Center Hamburg-Eppendorf, Hamburg, Germany; 18 Saarland Cancer Registry, Saarbrücken, Germany; 19 Institute for Prevention and Cancer Epidemiology, Faculty of Medicine and Medical Center, University of Freiburg, Freiburg, Germany; 20 Division of Cancer Epidemiology, German Cancer Research Center (DKFZ), Heidelberg, Germany; 21 Institute of Social Medicine, Epidemiology and Health Economics, Charité - Universitätsmedizin Berlin, Berlin, Germany; 22 Institute for Clinical Epidemiology and Biometry, University of Würzburg, Würzburg, Germany; 23 State Institute of Health, Bavarian Health and Food Safety Authority, Erlangen, Germany; 24 Institute for Epidemiology and Preventive Medicine, University of Regensburg, Regensburg, Germany; 25 Leipzig Research Centre for Civilization Diseases (LIFE), University of Leipzig, Leipzig, Germany; 26 Institute for Community Medicine, University Medicine Greifswald, Greifswald, Germany; 27 IUF - Leibniz Research Institute for Environmental Medicine, Düsseldorf, Germany; 28 Institute of Epidemiology, Helmholtz Zentrum München - German Research Center for Environmental Health, Neuherberg, Germany; 29 Molecular Epidemiology Research Group, Max-Delbrück-Center for Molecular Medicine in the Helmholtz Association (MDC), Berlin, Germany

**Keywords:** Intersectionality, Social inequality, Cohort study, Study participants

## Abstract

**Background:**

Intersectionality has contributed to novel insights in epidemiology. However, participants of epidemiological studies have rarely been characterised from an intersectional perspective. We aimed to show the gained insights of an intersectionality-informed approach to describing a study population by comparing it to a conventional approach.

**Methods:**

We used data of the German National Cohort (NAKO), which recruited 205,415 participants between 2014 and 2019. In the conventional approach, marginal proportions of educational level, cohabitation status, and country of birth were compared between the study populations of the NAKO and the German census survey (MZ) of 2014. In the intersectionality-informed approach, so-called intersectional population strata were constructed by cross-classifying educational level, cohabitation status, and country of birth. Proportions of these strata were also compared between NAKO and MZ. All analyses were stratified by sex and age group.

**Results:**

The conventional approach showed that the proportion of people with low education was lower in the NAKO compared to the MZ in all sex and age strata. Similarly, proportions of all intersectional population strata with low education were lower in the NAKO. Concerning cohabitation, the conventional approach showed that the proportion of those living without a partner was lower in the NAKO than in the MZ for women under 60 and men. The intersectionality-informed approach revealed that the proportions of some subgroups of those living without a partner were higher in the NAKO than in the MZ. These were intersectional population strata who lived without a partner, had a high level of education and were born in Germany. The intersectionality-informed approach revealed similar within-group heterogeneity for country of birth, showing that not all proportions of foreign-born people were lower in the NAKO compared to the MZ. Proportions of foreign-born with high education who lived with a partner were higher.

**Conclusions:**

Our results showed that heterogeneity within social categories can be revealed by applying the concept of intersectionality when comparing study participants with an external population. This way, an intersectionality-informed approach contributes to describing social complexity among study participants more precisely. Furthermore, results can be used to reduce participation barriers in a more targeted way.

**Supplementary Information:**

The online version contains supplementary material available at 10.1186/s12939-025-02521-3.

## Background

Intersectionality has gained attention in epidemiology and public health in recent years [1–4]. The theoretical concept, which was developed in Black Feminism and Critical Race Theory, generally posits that social categories such as sex/gender, ethnic origin, socioeconomic position, social support, or age are socially constructed and that their interactions have to be considered in analyses of social inequality [5–8]. Furthermore, intersectionality emphasises that social inequality needs to be understood against the backdrop of historically rooted power relations [5–8]. Further theoretical tenets, the explanation of which would exceed the scope of this text, have been published elsewhere [5–8]. Crenshaw was among the first to illustrate that an intersectional perspective is necessary to understand social inequality. She demonstrated in an analysis of legal cases that discrimination against Black women in the United States of America (USA) cannot be explained by sexism, racism or their addition. Instead, Black women are affected by a unique form of discrimination [6]. In epidemiology, first empirical studies have highlighted the insights of an intersectionality-informed perspective. For example, a study of the USA adult population showed that low income Black females had a higher mean BMI than would be expected from the additive main effects of low income, female sex, and Black identity [9]. Another study found gender and race bias in referrals for cardiac catheterisation exclusively among Black females, while Black males, white females, and white males were unaffected [10]. These empirical results are accompanied by important methodological developments that allow the integration of some tenets of intersectionality in epidemiological research methods. For example, intersectionality-informed approaches for measurement and statistical analysis have been developed [9, 11–14].

Against the backdrop of these advancements, it is surprising that participants in epidemiological studies have rarely been characterised from an intersectional perspective [15]. Currently, it is not known to which degree the intersectional profile of general populations compares to the intersectional profile of study populations in large population-based cohorts. To describe the intersectional profile of a population, social categories should be cross-classified to yield so-called intersectional population strata [15]. This approach may uncover within-group heterogeneity that remains hidden when analysing marginal distributions of single social categories independently. For example, past research suggested that the proportion of females in observational studies is larger than in the corresponding general populations [16–21]. A review comparing results of studies that stratified the group of females by further social categories, however, found that the proportion of females under 35 years, with Asian ethnicity, without school-leaving degree, and with low income was often lower than in the general population [22].

Moreover, it is crucial for intersectionality-informed epidemiological research that sufficient numbers of observations are available within intersectional population strata in order to conduct analyses on subgroup-specific causes of good and ill health. Such analyses may contribute to a better understanding of effect heterogeneity in a population [23]. Oversampling intersectional population strata may be an effective method to enable subgroup analyses. On the other hand, population-based cohort studies, such as the German National Cohort (NAKO Gesundheitsstudie), provide another unique opportunity since intersectional population strata may include sufficient numbers of observations due to the very large sample size [24].

The primary aim of this study was to conduct an intersectionality-informed description of participants in the NAKO and to compare results of this approach with results from a conventional analysis. In the conventional approach, we compared marginal distributions of educational level, cohabitation status, and country of birth between the NAKO and a German census survey, stratified by sex and age group. In the intersectionality-informed approach, we cross-classified educational level, cohabitation status, and country of birth to construct intersectional population strata and compared the distribution of these subgroups between the NAKO and the census survey within the same sex and age strata. This way, we aimed to describe the within-group heterogeneity that is revealed by the intersectionality-informed compared to the conventional perspective. The secondary aim of the study was to calculate numbers of observations for all constructed intersectional population strata with the aim to identify promising starting points for future subgroup analyses in the NAKO.

## Methods

### Study design and population

NAKO is a population-based cohort study that enrolled 205,415 participants between 2014 and 2019. Eighteen study centers of the NAKO are located in 16 study regions throughout Germany. The study population was selected based on random samples drawn from compulsory population registries in the respective study regions from persons between 20 and 69 years of age. Furthermore, sampling was stratified by sex and 10-year age groups. More specifically, the study design aimed to include 10,000 participants in each 10-year age group between 20 and 39 years, and 26,667 participants in each 10-year age group between 40 and 69 years for females and males. Details of the study design have been published elsewhere [24]. In this study, we used data of N = 200,471 observations after excluding participants who were below 20 years old or above 69 at the baseline interview, as well as those who withdrew consent. We used sociodemographic data from baseline that were collected in standardised, computer-assisted face-to-face interviews. Internal quality management was conducted by the NAKO consortium and an external quality control was performed by the Robert Koch-Institute, Germany [24]. To compare social categories of the NAKO study population with the general German population, we used the scientific use file of the Mikrozensus (MZ) survey from 2014 [25]. The MZ is a compulsory census survey and representative for the general German population. The sample is drawn based on a single stage clustered sampling design. Sampling units are artificial selection districts, in which all individuals and households are surveyed. In the survey of 2014, the non-response proportion for eligible households was 2.4%. We used data from all MZ participants aged 20 to 69 years (N = 310,832). Data collection of the MZ was conducted applying computer-assisted personal interviews (CAPI) [26].

## Assessment of variables

Variables from five social categories were selected from both studies. We used only variables that were defined using the same operationalisations in the NAKO and the MZ. Both studies collected information on sex (female or male) and age. The variable educational level was selected from the category socioeconomic position [27]. Educational level was operationalised using information on the highest general school-leaving certificate of the German schooling system. In both the NAKO and the MZ, foreign school-leaving degrees had to be assigned to the best matching certificate of the German schooling system. Current students without certificate, school-leavers without certificate, and all degrees that were lower or equivalent to the type “Hauptschulabschluss (Volksschulabschluss)”, which corresponds to a degree after the 9 th grade, were classified as low educational level. A degree from the “Polytechnische Oberschule der DDR” (Polytechnic Secondary School of the German Democratic Republic) up to grade 9 was also classified as low educational level. A medium level of education was defined as the degree “Mittlere Reife” or a final degree from the “Polytechnische Oberschule der DDR”, which corresponds in both cases to a degree after the 10 th grade. All certificates higher than those included in the medium level were defined as high level (“Fachhochschulreife”, “Abitur”). The variable cohabitation was selected from the category social support [28]. Cohabitation was a binary variable with the two categories “living with a partner” and “not living with a partner”. Finally, country of birth was selected from the social category ethnic origin [29]. This variable was also binary and delineated the groups of people born in Germany from people not born in Germany.

## Statistical Methods

Due to the study design of the NAKO, we performed all analyses stratified by sex and 10-year age group. Participants with missing values in educational level, cohabitation, or country of birth were excluded in all analyses. We present only results for the age groups 20–29, 40–49, and 60–69 years, because we aimed to highlight the gained insights of an intersectionality-informed approach as an example. Results of the other age groups, which contain no unexpected outliers and can be derived from the general age gradient among both sexes, are shown in Additional File 1. In the first step, a “conventional” analysis was performed by the current practice of describing study populations in epidemiology [19, 20, 30, 31]. In this approach, marginal proportions were calculated for educational level, cohabitation, and country of birth together with 95% confidence intervals (95% CI). When calculating proportions in the MZ, we considered the clustered sampling design and applied weights to account for non-response. In the second step, we applied an “intersectionality-informed” approach. To implement this approach, intersectional population strata were constructed by cross-classifying the variables educational level, cohabitation, and country of birth which resulted in 3 × 2x2 = 12 intersectional population strata within each sex and age stratum. Proportions together with 95% CIs were calculated for each constructed intersectional stratum in the same way as in the conventional analysis. Confidence intervals are only shown in Additional File 1. Results of the conventional approach were compared with results of the intersectionality-informed approach separately for level of education, cohabitation, and country of birth. Finally, we calculated numbers of participants for each intersectional stratum, stratified by sex and by the two age groups 20–39 and 40–69 years.

## Results

Among all participants of the NAKO, 50.5% were female, 15.8% had a low level of education, 29.4% were living alone, and 11.7% were born outside Germany (Table 1). In the MZ, 50.4% were female, 33.2% had a low level of education, 37.0% were living alone, and 16.0% were born outside Germany. Among all included participants from the NAKO, N = 962 (0.5%) participants had missing values in either educational level, cohabitation or country of birth, while N = 676 (0.2%) of the included participants from the MZ had missing values in educational level.

**Table 1:** Descriptive characteristics of the study populations of the German National Cohort (years of recruitment 2014–2019) and the Mikrozensus (year of recruitment 2014)

	**NAKO**	**MZ**
	**(N = 200,471)**	**(N = 310,832)**
**Sex**		
Female	101,264 (50.5%)	156,634 (50.4%)
Male	99,207 (49.5%)	154,198 (49.6%)
Missing	0 (0.0%)	0 (0.0%)
**Age group**		
20–29	19,958 (10.0%)	53,653 (17.3%)
30–39	21,652 (10.8%)	54,741 (17.6%)
40–49	53,073 (26.5%)	70,481 (22.7%)
50–59	54,962 (27.4%)	75,039 (24.1%)
60–69	50,826 (25.4%)	56,918 (18.3%)
missing	0 (0.0%)	0 (0.0%)
**Level of education**		
High	105,253 (52.5%)	106,062 (34.1%)
Medium	62,945 (31.4%)	100,972 (32.5%)
Low	31,586 (15.8%)	103,122 (33.2%)
Missing	687 (0.3%)	676 (0.2%)
**Cohabitation**		
With partner	141,104 (70.4%)	195,923 (63.0%)
Without partner	58,955 (29.4%)	114,909 (37.0%)
Missing	412 (0.2%)	0 (0.0%)
**Country of birth**		
Germany	176,834 (88.2%)	261,145 (84.0%)
Born abroad	23,495 (11.7%)	49,687 (16.0%)
Missing	142 (0.1%)	0 (0.0%)

NAKO: German National Cohort

MZ: Mikrozensus, annual census survey of Germany

In the conventional description of educational level, the proportion of those with high education was higher in the NAKO compared to the MZ for all sex and age strata (Table 2). In the intersectionality-informed approach showed that the proportion of one subgroup with high education was lower among the NAKO study population compared with the general German population. This was the intersectional population stratum with high education, living without a partner and with a country of birth outside Germany among males aged 20–29 years. Considering results for NAKO participants with medium education, the conventional approach showed that their proportion was lower compared to the proportion in the MZ in the age groups 20–29 and 40–49, and higher in the age group 60–69 irrespective of sex. For the age groups 20–29 and 40–49, the intersectionality-informed approach revealed that the proportion of males with medium education, living with a partner, and Germany as a country of birth was higher in the NAKO compared to the MZ. For the age group 60–69, the intersectional perspective showed that the proportion of males with medium education, living with a partner, and a country of birth outside Germany was lower in the NAKO compared to the MZ. Finally, in the conventional approach, proportions of people with low education were lower in the NAKO compared to the MZ. Similarly, proportions of all intersectional population strata with low education were lower in the NAKO compared to the MZ.

**Table 2:** Level of education among participants of the German National Cohort compared with participants of the Mikrozensus (conventional vs. intersectionality-informed approach)

			**Female**						**Male**					
			**20–29 years**		**40–49 years**		**60–69 years**		**20–29 years**		**40–49 years**		**60–69 years**	
			NAKO	MZ	NAKO	MZ	NAKO	MZ	NAKO	MZ	NAKO	MZ	NAKO	MZ
			%	%	%	%	%	%	%	%	%	%	%	%
Conventional approach														
**Level of education**														
High			78.6⁺	52.7	54.3⁺	32.3	35.3⁺	16.8	75.7⁺	45.8	56.8⁺	34.1	45.1⁺	27.1
Medium			17.6⁻	29.8	36.6⁻	41.5	36.9⁺	28.6	18.4⁻	29.0	30.1⁻	32.8	27.1⁺	22.1
Low			3.8⁻	17.5	9.1⁻	26.2	27.8⁻	54.7	5.9⁻	25.2	13.1⁻	33.2	27.7⁻	50.8
Intersectionality-informed approach														
** Level of education**	**Cohabitation**	**Country of birth**												
High	with partner	Germany	29.8⁺	12.8	33.2⁺	18.4	20.0⁺	8.9	24.4⁺	7.2	38.5⁺	20.6	33.3⁺	18.7
High	with partner	Born abroad	3.4	3.5	6.2⁺	4.4	3.0⁺	2.2	2.3	1.9	5.9⁺	3.6	3.4⁺	2.7
High	without partner	Germany	41.9⁺	32.1	12.8⁺	7.9	10.4⁺	4.5	44.9⁺	31.7	10.9⁺	8.5	7.5⁺	4.7
High	without partner	Born abroad	3.5	4.2	2.1⁺	1.6	1.8⁺	1.1	4.1⁻	5.0	1.5	1.3	0.9	1.0
Medium	with partner	Germany	9.7	10.7	24.3⁻	26.5	24.1⁺	18.6	7.4⁺	6.4	20.3⁺	19.2	21⁺	15.9
Medium	with partner	Born abroad	0.5⁻	2.1	2.7⁻	3.7	1.5	1.7	0.5⁻	1.1	2.5⁻	3.4	1.5⁻	1.9
Medium	without partner	Germany	6.8⁻	15.3	8.8⁻	10.2	10.5⁺	7.4	9.8⁻	19.6	6.8⁻	9.3	4.2	3.9
Medium	without partner	Born abroad	0.5⁻	1.6	0.9	1.1	0.9	0.9	0.7⁻	1.9	0.5⁻	0.9	0.4	0.3
Low	with partner	Germany	1.6⁻	4.9	4.7⁻	12.1	17.4⁻	32.3	1.7⁻	4.6	6.7⁻	14.5	20.0⁻	31.9
Low	with partner	Born abroad	0.5⁻	2.8	2.0⁻	6.3	1.7⁻	6.0	0.5⁻	1.7	2.7⁻	6.7	2.4⁻	7.0
Low	without partner	Germany	1.4⁻	8.2	1.6⁻	5.9	7.7⁻	13.6	3.1⁻	15.6	2.9⁻	9.7	4.7⁻	10.3
Low	without partner	Born abroad	0.3⁻	1.6	0.7⁻	1.8	1.0⁻	2.8	0.7⁻	3.3	0.7⁻	2.2	0.6⁻	1.6

NAKO: German National Cohort; MZ: Mikrozensus survey

⁺ confidence interval does not overlap with confidence interval of corresponding proportion from MZ, proportion in NAKO higher than in MZ

⁻ confidence interval does not overlap with confidence interval of corresponding proportion from MZ, proportion in NAKO lower than in MZ

Turning to the conventional analysis of cohabitation status, among females under 60 and males, the proportion of people living without a partner was lower in the NAKO compared to the MZ (Table 3). The proportion of females aged 60–69 living alone was higher in the NAKO compared to the MZ. The intersectionality-informed approach showed that the proportion of those living without a partner, with a high level of education, and with Germany as a country of birth was higher among females and males in the age groups 20–29 and 40–49. In addition, among females aged 40–49 years, the proportion of those at the intersection of not cohabiting with a partner, a high education, and a country of birth outside Germany was also higher.

**Table 3:** Cohabitation among participants of the German National Cohort compared with participants of the Mikrozensus (conventional vs. intersectionality-informed approach)

			**Female**						**Male**					
			**20–29 years**		**40–49 years**		**60–69 years**		**20–29 years**		**40–49 years**		**60–69 years**	
			NAKO	MZ	NAKO	MZ	NAKO	MZ	NAKO	MZ	NAKO	MZ	NAKO	MZ
			%	%	%	%	%	%	%	%	%	%	%	%
Conventional approach														
**Cohabitation**														
with partner			45.6⁺	36.9	73.1⁺	71.5	67.7⁻	69.7	36.7⁺	22.8	76.7⁺	68.0	81.7⁺	78.2
without partner			54.4⁻	63.1	26.9⁻	28.5	32.3⁺	30.3	63.3⁻	77.2	23.3⁻	32.0	18.3⁻	21.8
Intersectionality-informed approach														
**Cohabitation**	**Level of education**	**Country of birth**												
with partner	High	Germany	29.8⁺	12.8	33.2⁺	18.4	20.0⁺	8.9	24.4⁺	7.2	38.5⁺	20.6	33.3⁺	18.7
with partner	High	Born abroad	3.4	3.5	6.2⁺	4.4	3.0⁺	2.2	2.3	1.9	5.9⁺	3.6	3.4⁺	2.7
with partner	Medium	Germany	9.7	10.7	24.3⁻	26.5	24.1⁺	18.6	7.4⁺	6.4	20.3⁺	19.2	21.0⁺	15.9
with partner	Medium	Born abroad	0.5⁻	2.1	2.7⁻	3.7	1.5	1.7	0.5⁻	1.1	2.5⁻	3.4	1.5⁻	1.9
with partner	Low	Germany	1.6⁻	4.9	4.7⁻	12.1	17.4⁻	32.3	1.7⁻	4.6	6.7⁻	14.5	20.0⁻	31.9
with partner	Low	Born abroad	0.5⁻	2.8	2.0⁻	6.3	1.7⁻	6.0	0.5⁻	1.7	2.7⁻	6.7	2.4⁻	7.0
without partner	High	Germany	41.9⁺	32.1	12.8⁺	7.9	10.4⁺	4.5	44.9⁺	31.7	10.9⁺	8.5	7.5⁺	4.7
without partner	High	Born abroad	3.5	4.2	2.1⁺	1.6	1.8⁺	1.1	4.1⁻	5.0	1.5	1.3	0.9	1.0
without partner	Medium	Germany	6.8⁻	15.3	8.8⁻	10.2	10.5⁺	7.4	9.8⁻	19.6	6.8⁻	9.3	4.2	3.9
without partner	Medium	Born abroad	0.5⁻	1.6	0.9	1.1	0.9	0.9	0.7⁻	1.9	0.5⁻	0.9	0.4	0.3
without partner	Low	Germany	1.4⁻	8.2	1.6⁻	5.9	7.7⁻	13.6	3.1⁻	15.6	2.9⁻	9.7	4.7⁻	10.3
without partner	Low	Born abroad	0.3⁻	1.6	0.7⁻	1.8	1.0⁻	2.8	0.7⁻	3.3	0.7⁻	2.2	0.6⁻	1.6

NAKO: German National Cohort; MZ: Mikrozensus survey

⁺ confidence interval does not overlap with confidence interval of corresponding proportion from MZ, proportion in NAKO higher than in MZ

⁻ confidence interval does not overlap with confidence interval of corresponding proportion from MZ, proportion in NAKO lower than in MZ

Finally, the conventional approach showed that the proportion of people born abroad was lower in the NAKO compared to the MZ across all sex and age strata (Table 4). The intersectionality-informed approach, in contrast, revealed that proportions of some intersectional population strata with a country of birth outside Germany were higher. Among males aged 20–29 years, the proportion of those at the intersection of a country of birth outside Germany, a high education, and a cohabitation with a partner was higher. In addition, among females and males aged 40–49 or 60–69 years, the proportion of those at the intersection of a country of birth outside Germany, a high education, and a cohabitation with a partner was higher. Finally, among females aged 40–49 or 60–69 years, the proportion of those at the intersection of a country of birth outside Germany, a high education, and no cohabitation with a partner was also higher compared to the MZ.

**Table 4:** Country of birth among participants of the German National Cohort compared with participants of the Mikrozensus (conventional vs. intersectionality-informed approach)

			**Female**						**Male**					
			**20–29 years**		**40–49 years**		**60–69 years**		**20–29 years**		**40–49 years**		**60–69 years**	
			NAKO	MZ	NAKO	MZ	NAKO	MZ	NAKO	MZ	NAKO	MZ	NAKO	MZ
			%	%	%	%	%	%	%	%	%	%	%	%
Conventional approach														
**Country of birth**														
Germany			91.3⁺	84.1	85.5⁺	81.0	90.2⁺	85.2	91.2⁺	85.1	86.1⁺	81.8	90.8⁺	85.4
Born abroad			8.7⁻	15.9	14.5⁻	19.0	9.8⁻	14.8	8.8⁻	14.9	13.9⁻	18.2	9.2⁻	14.6
Intersectionality-informed approach														
**Country of birth**	**Level of education**	**Cohabitation**												
Germany	High	with partner	29.8⁺	12.8	33.2⁺	18.4	20.0⁺	8.9	24.4⁺	7.2	38.5⁺	20.6	33.3⁺	18.7
Germany	High	without partner	41.9⁺	32.1	12.8⁺	7.9	10.4⁺	4.5	44.9⁺	31.7	10.9⁺	8.5	7.5⁺	4.7
Germany	Medium	with partner	9.7	10.7	24.3⁻	26.5	24.1⁺	18.6	7.4⁺	6.4	20.3⁺	19.2	21.0⁺	15.9
Germany	Medium	without partner	6.8⁻	15.3	8.8⁻	10.2	10.5	7.4	9.8⁻	19.6	6.8⁻	9.3	4.2	3.9
Germany	Low	with partner	1.6⁻	4.9	4.7⁻	12.1	17.4⁻	32.3	1.7⁻	4.6	6.7⁻	14.5	20.0⁻	31.9
Germany	Low	without partner	1.4⁻	8.2	1.6⁻	5.9	7.7⁻	13.6	3.1⁻	15.6	2.9⁻	9.7	4.7⁻	10.3
Born abroad	High	with partner	3.4	3.5	6.2⁺	4.4	3.0⁺	2.2	2.3	1.9	5.9⁺	3.6	3.4⁺	2.7
Born abroad	High	without partner	3.5	4.2	2.1⁺	1.6	1.8⁺	1.1	4.1⁻	5.0	1.5	1.3	0.9	1.0
Born abroad	Medium	with partner	0.5⁻	2.1	2.7⁻	3.7	1.5	1.7	0.5⁻	1.1	2.5⁻	3.4	1.5⁻	1.9
Born abroad	Medium	without partner	0.5⁻	1.6	0.9	1.1	0.9	0.9	0.7⁻	1.9	0.5⁻	0.9	0.4	0.3
Born abroad	Low	with partner	0.5⁻	2.8	2.0⁻	6.3	1.7⁻	6.0	0.5⁻	1.7	2.7⁻	6.7	2.4⁻	7.0
Born abroad	Low	without partner	0.3⁻	1.6	0.7⁻	1.8	1.0⁻	2.8	0.7⁻	3.3	0.7⁻	2.2	0.6⁻	1.6

NAKO: German National Cohort; MZ: Mikrozensus survey

⁺ confidence interval does not overlap with confidence interval of corresponding proportion from MZ, proportion in NAKO higher than in MZ

⁻ confidence interval does not overlap with confidence interval of corresponding proportion from MZ, proportion in NAKO lower than in MZ

Finally, Table 5 shows numbers of participants in the NAKO, stratified by sex and a binary age group. In total there were 12 x 2 x 2 = 48 strata that could be assessed using this table. Among both females and males in the age group 20–39 years, over 60% of participants were people with high education who were born in Germany. The stratum with the lowest number of participants (N = 87) were females with low education, living alone, and with a country of birth outside Germany. The stratum with most participants (N = 7,515) were females with high education, living with a partner and with Germany as country of birth. In the age group 40–69 years, participants were distributed more evenly across levels of education compared to the age group 20–39 years. Over 50% of participants were people with high or medium education, living with a partner, and born in Germany. Here, the stratum with the lowest number of participants (N = 341) were males with medium education, living alone and being born outside Germany. Most participants (N = 27,373) were males with high education, living with a partner and with Germany as country of birth.

**Table 5:** Numbers of participants in the German National Cohort for intersectional population strata, stratified by sex and by the age groups 20–39 and 40–69 years

			**Female**		**Male**	
			N	%	N	%
			**20–39 years**			
**education**	**cohabitation**	**country of birth**				
high	with partner	Germany	7515	35.7	6611	32.4
high	with partner	abroad	1130	5.4	925	4.5
high	w/o partner	Germany	6182	29.4	6177	30.3
high	w/o partner	abroad	648	3.1	696	3.4
medium	with partner	Germany	2718	12.9	2390	11.7
medium	with partner	abroad	278	1.3	240	1.2
medium	w/o partner	Germany	1344	6.4	1615	7.9
medium	w/o partner	abroad	127	0.6	120	0.6
low	with partner	Germany	492	2.3	661	3.2
low	with partner	abroad	201	1.0	249	1.2
low	w/o partner	Germany	299	1.4	586	2.9
low	w/o partner	abroad	87	0.4	129	0.6
			**40–69 years**			
**education**	**cohabitation**	**country of birth**				
high	with partner	Germany	21,389	26.8	27,373	35.0
high	with partner	abroad	3474	4.4	3472	4.4
high	w/o partner	Germany	9602	12.0	7431	9.5
high	w/o partner	abroad	1505	1.9	956	1.2
medium	with partner	Germany	20,165	25.3	16,939	21.6
medium	with partner	abroad	1596	2.0	1522	1.9
medium	w/o partner	Germany	8018	10.0	4782	6.1
medium	w/o partner	abroad	681	0.9	341	0.4
low	with partner	Germany	7967	10.0	9924	12.7
low	with partner	abroad	1468	1.8	2041	2.6
low	w/o partner	Germany	3283	4.1	3006	3.8
low	w/o partner	abroad	634	0.8	520	0.7

## Discussion

Our study showed that an intersectionality-informed approach reveals within-group heterogeneity when comparing participants of a large population-based cohort study with the general population. For example, the conventional approach suggested that the proportion of participants who were born abroad was lower in the NAKO compared to the MZ for all sex and age strata. In the intersectionality-informed approach, on the other hand, not all strata with a country of birth outside Germany showed a lower proportion than in the MZ. Such within-group heterogeneity was also shown for level of education and cohabitation when assessing intersectional population strata. Finally, we found that the numbers of participants in important intersectional population strata may allow to study effect heterogeneity in future research. For example, there were over 500 observations in the intersectional population stratum of people aged 40–69 years with low education, living alone and a country of birth outside Germany for women and men, respectively.

A previous review found that no intersectionality-informed descriptions of study populations in epidemiological cohort studies have been carried out to date [15]. Furthermore, non-response in a national health survey was assessed from an intersectional perspective. Findings from this study are similar to the results presented in this analysis, since response proportions were lowest for people at the intersection of not being married and a low level of education [32]. Moreover, an occupational cohort study from France investigated interaction of three social categories in their analysis of study participation. They showed that, in males, high age was associated with lower study participation exclusively among unskilled workers [31]. Among females, in contrast, high age was associated with lower participation only among managers [31]. These results point out that multiple social categories may interact when being assessed for association with study participation. An intersectionality-informed perspective contributes to make these interactions visible.

Turning to the strengths and limitations of our study, the large sample size of the NAKO and the MZ resulted in a high precision of the calculated proportions for most intersectional population strata. This feature allowed meaningful comparisons of subgroups that make up rather small proportions of the general German population. Concerning statistical methods, cross-classification is a simple approach that is suited for intersectionality-informed quantitative analyses of studies with more than 200.000 observations [11]. In addition, education, cohabitation, and country of birth were defined in the NAKO and the MZ using the same operationalization, making direct comparisons possible. However, the need to use comparable variables gave rise to the limitation that only limited information on social categories could be compared. More detail is generally warranted in intersectionality-informed research [4]. For example, further dimensions of social inequality such as occupational class, income, place of residence, religion, or disability are of high interest [33]. Moreover, we could only use information on sex, while variables on gender (e.g. gender identity, gender roles, or perceived inequality in the partnership) were not available. In addition, country of birth represents just a fraction of characteristics that are grounds for racial and ethnic discrimination [34]. People can also be subject to discrimination if they speak a language that is not German or with a certain accent, if they are racified based on perceived appearance, or if they identify with an ethnic group [35]. In addition, not all people born outside Germany are affected by racial or ethnic discrimination within Germany. Hence, considerable heterogeneity remains within the intersectional population strata used in our analysis. Finally, due to the descriptive nature of our study, we were not able to assess causes of the observed differences. Despite these limitations, our study provides interpretable results and offers a starting point to uncovering within-group heterogeneity of study populations from an intersectional perspective.

It is important to note that conclusions on representativeness of the NAKO for the German general population are not possible, because the study is performed in 18 study centers that were not randomly selected. Moreover, the respective local regulations at the study centers led to slightly different ways to arrive at the final population sample, and therefore, weights have been calculated for each center separately. The underlying weighting strategy of the NAKO, which is currently being developed, does not target Germany as a whole, but the target populations of the individual study regions [36]. What is more, estimating measures of effect in the total sample of a representative population-based study is usually driven by the privileged majority and may not provide a good estimate for all population subgroups [37]. An intersectionality-informed perspective rather supports an increased focus on subgroup analyses and studies of effect heterogeneity [1]. Past research has identified several causal relationships where effect heterogeneity across social categories may exist. For example, a large multicentre trial showed that captopril, an oral angiotensin-converting-enzyme (ACE) inhibitor, is more effective in the treatment of mild to moderate hypertension among white people in comparison to Black people [38]. Moreover, responses to lung cancer immunotherapy differ between females and males according to a meta-analysis [39]. In addition, an observational study suggested that the availability of green spaces is associated with better subjective health among people with low education but not among people with high education [40]. Sufficient numbers of observations are needed within each subgroup to study effect heterogeneity with high statistical power. In a representative sampling strategy, this goal may not be achieved for some intersectional population strata, if their proportion in the general population is low. Rather, oversampling based on pre-specified power calculations should be applied. Finally, to study effect heterogeneity, a strong theoretical rationale that justifies the selection of subgroups is warranted, since testing for interaction may quickly lead to a large type-I error rate. Intersectionality provides such a theoretical rationale, because intersectional population strata are assumed to experience unique living conditions that may act as effect measure modifiers [1, 12].

Our study has shown that large cohorts such as the NAKO provide an important resource for intersectionality-informed research, since there may be sufficient numbers of observations for many intersectional population strata to conduct meaningful studies on effect heterogeneity. In the age group 40–69 of the NAKO, for example, there were over 500 participants in the intersectional population stratum of people with a low education, living without partner, and being born outside Germany for females and males, respectively. This population stratum makes up a small fraction in the general population, but is of high importance from an intersectional perspective, because it combines three characteristics that are related to a position of relatively low societal power. For many research questions, it may be adequate to cross-classify fewer variables than in our study, leaving even more observations that can be used for interaction or subgroup analysis. For strata with insufficient numbers of observations, measures to reduce participation barriers should be developed. This endeavour is challenging, since response proportions have been declining in the past and study populations are often healthier, better educated, and wealthier compared to the general populations [19, 20, 24]. Existing research points out starting points to reduce barriers for participation. For example, an intersectionality-informed qualitative investigation among participants and non-participants suggested that time constraints due to care responsibilities limit possibility to take part in a time-intensive examination program [41]. Further research suggested that a lack of transportation, interference with work/family responsibilities, financial costs, and burdensome procedures to be important barriers to participation [42]. Moreover, home visits, multilingual explanation videos and video-interpretation services may increase participation in epidemiological studies among people with migration background [43]. Since participants who were difficult to recruit initially are more likely to drop out at later stages of a study, special efforts might also be necessary to keep such groups enrolled in a study [44]. However, information on study participation and associated barriers among intersectional population strata is still limited, mainly because non-response studies are inherently hampered by the lack of information on non-respondents. Hence, to understand the intersectional nature of non-response in population-based research and to adequately address its causes, studies are needed that go beyond conventional non-response analyses and specifically take an intersectional perspective.

## Conclusions

To conclude, thoroughly describing the social complexity of study populations from an intersectional perspective may aid to avoid false universalism [45]. False universalism describes the unjustified and often implicit assumption that a single social group serves as the model human for scientific inquiry and does not portray any social heterogeneity in the important discourse about the causes of health and disease [46]. Finally, large cohort studies should be used more often in intersectionality-informed research, since sample size of intersectional population strata with small proportions in the general population may be sufficient to conduct meaningful subgroup analyses. These analyses may contribute more information on effect heterogeneity within human populations. For subgroups with few numbers of observations, future research should focus on elucidating specific participation barriers and suggest measures to reduce them.

## Supplementary Information

Additional file 1: Supplementary Table 1. Comparison of education, cohabitation, and country of birth between the NAKO and the MZ, stratified by sex and age group. Supplementary Table 2. Comparison of the intersectional population strata between the NAKO and the MZ, stratified by sex and age group

## Data Availability

The data that support the findings of this study are available from the German National Cohort (NAKO) but restrictions apply to the availability of these data, which were used under license for the current study, and so are not publicly available. Data are however available from the authors upon reasonable request and with permission of NAKO.

## Abbreviations

ACE: Angiotensin-converting-enzyme
CAPI: Computer-assisted personal interview
CI: Confidence interval
DDR: German Democratic Republic
MZ: German census survey - Mikrozensus
NAKO: German National Cohort
USA: United States of America
