# Supplementary material for: What can we learn from an intersectionality-informed description of study participants? Results from the German National Cohort
Source: Int J Equity Health. 2025 May 26;24:151. doi: 10.1186/s12939-025-02521-3 (PMC12107990; doi:10.1186/s12939-025-02521-3)
Supplement: Supplementary file 1 — Additional file 1: Supplementary Table 1. Comparison of education, cohabitation, and country of birth between the NAKO and the MZ, stratified by sex and age group. Supplementary Table 2. Comparison of the intersectional population strata between the NAKO and the MZ, stratified by sex and age group [file 12939_2025_2521_MOESM1_ESM.pdf]

What can we learn from an intersectionality-informed description of study participants? Results from the German National Cohort

Additional File 1, Supplementary Table 1: Comparison of education, cohabitation, and country of birth between the NAKO and the MZ, stratified by sex and age group. (page 2)

Additional File 1, Supplementary Table 2: Comparison of the intersectional population strata between the NAKO and the MZ, stratified by sex and age group. (page 4)

Supplementary Table 1: Comparison of education, cohabitation, and country of birth between the NAKO and the MZ, stratified by sex and age group.

	Female												Male											
	NAKO						MZ						NAKO						MZ					
	Prop. %	95% CI					Prop. %	95% CI					Prop. %	95% CI					Prop. %	95% CI				
**20-29 years**																								
Level of education																								
High	78.6	77.8	-	79.4			52.7	51.9	-	53.4			75.7	74.8	-	76.5			45.8	45.0	-	46.6		
Medium	17.6	16.8	-	18.3			29.8	29.2	-	30.4			18.4	17.7	-	19.2			29.0	28.4	-	29.6		
Low	3.8	3.4	-	4.2			17.5	17.0	-	18.0			5.9	5.4	-	6.4			25.2	24.6	-	25.8		
Cohabitation																								
Living with partner	45.6	44.6	-	46.5			36.9	36.3	-	37.6			36.7	35.8	-	37.7			22.8	22.3	-	23.3		
Living w/o partner	54.4	53.5	-	55.4			63.1	62.4	-	63.7			63.3	62.3	-	64.2			77.2	76.6	-	77.7		
Country of birth																								
Born in Germany	91.3	90.8	-	91.8			84.1	83.6	-	84.6			91.2	90.7	-	91.8			85.1	84.5	-	85.6		
Born abroad	8.7	8.1	-	9.3			15.9	15.4	-	16.4			8.8	8.2	-	9.3			14.9	14.4	-	15.5		
**30-39 years**																								
Level of education																								
High	69.0	68.1	-	69.8			44.7	44.1	-	45.4			66.0	65.1	-	66.9			41.4	40.8	-	42.1		
Medium	24.6	23.8	-	25.5			34.0	33.4	-	34.6			24.1	23.3	-	25.0			30.1	29.5	-	30.6		
Low	6.4	5.9	-	6.9			21.2	20.7	-	21.8			9.9	9.3	-	10.4			28.5	27.9	-	29.1		
Cohabitation																								
Living with partner	70.8	69.9	-	71.6			68.9	68.3	-	69.5			70.5	69.6	-	71.3			58.7	58.0	-	59.3		
Living w/o partner	29.2	28.4	-	30.1			31.1	30.5	-	31.7			29.5	28.7	-	30.4			41.3	40.7	-	42.0		
Country of birth																								
Born in Germany	85.4	84.7	-	86.1			75.8	75.2	-	76.3			85.9	85.2	-	86.5			78.0	77.4	-	78.6		
Born abroad	14.6	13.9	-	15.3			24.2	23.7	-	24.8			14.1	13.5	-	14.8			22.0	21.4	-	22.6		
**40-49 years**																								
Level of education																								
High	54.3	53.7	-	54.9			32.3	31.7	-	32.8			56.8	56.2	-	57.4			34.1	33.5	-	34.6		
Medium	36.6	36.1	-	37.2			41.5	41.0	-	42.1			30.1	29.6	-	30.7			32.8	32.2	-	33.3		
Low	9.1	8.8	-	9.5			26.2	25.7	-	26.7			13.1	12.7	-	13.5			33.2	32.6	-	33.7		
Cohabitation																								
Living with partner	73.1	72.6	-	73.6			71.5	71.0	-	72.0			76.7	76.2	-	77.2			68.0	67.5	-	68.6		
Living w/o partner	26.9	26.4	-	27.4			28.5	28.0	-	29.0			23.3	22.8	-	23.8			32.0	31.4	-	32.5		
Country of birth																								
Born in Germany	85.5	85.0	-	85.9			81.0	80.5	-	81.5			86.1	85.6	-	86.5			81.8	81.3	-	82.3		
Born abroad	14.5	14.1	-	15.0			19.0	18.5	-	19.5			13.9	13.5	-	14.4			18.2	17.7	-	18.7		

Supplemental table 1: (continued)

	Female				Male			
	**NAKO**		**MZ**		**NAKO**		**MZ**	
	Prop. %	95% CI	Prop. %	95% CI	Prop. %	95% CI	Prop. %	95% CI
				**50-59 years**				
Level of education								
High	45.3	44.7 - 45.9	25.4	25.0 - 25.9	48.2	47.6 - 48.8	29.6	29.1 - 30.2
Medium	40.8	40.2 - 41.4	39.3	38.8 - 39.9	32.9	32.4 - 33.4	29.8	29.3 - 30.4
Low	13.9	13.5 - 14.4	35.2	34.7 - 35.8	18.9	18.4 - 19.3	40.5	40.0 - 41.1
Cohabitation								
Living with partner	69.9	69.4 - 70.5	71.7	71.2 - 72.2	76.6	76.0 - 77.1	71.4	70.9 - 71.9
Living w/o partner	30.1	29.5 - 30.6	28.3	27.8 - 28.8	23.4	22.9 - 24.0	28.6	28.1 - 29.1
Country of birth								
Born in Germany	89.2	88.8 - 89.5	84.9	84.5 - 85.4	89.3	88.9 - 89.6	84.8	84.4 - 85.3
Born abroad	10.8	10.5 - 11.2	15.1	14.6 - 15.5	10.7	10.4 - 11.1	15.2	14.7 - 15.6
				**60-69 years**				
Level of education								
High	35.3	34.7 - 35.9	16.8	16.3 - 17.2	45.1	44.5 - 45.8	27.1	26.5 - 27.7
Medium	36.9	36.3 - 37.5	28.6	28.0 - 29.1	27.1	26.6 - 27.7	22.1	21.6 - 22.7
Low	27.8	27.2 - 28.3	54.7	54.0 - 55.3	27.7	27.2 - 28.3	50.8	50.1 - 51.5
Cohabitation								
Living with partner	67.7	67.1 - 68.3	69.7	69.1 - 70.2	81.7	81.2 - 82.1	78.2	77.6 - 78.7
Living w/o partner	32.3	31.7 - 32.9	30.3	29.8 - 30.9	18.3	17.9 - 18.8	21.8	21.3 - 22.4
Country of birth								
Born in Germany	90.2	89.8 - 90.6	85.2	84.7 - 85.7	90.8	90.5 - 91.2	85.4	84.9 - 85.9
Born abroad	9.8	9.4 - 10.2	14.8	14.3 - 15.3	9.2	8.8 - 9.5	14.6	14.1 - 15.1

Prop.: proportion, CI: confidence interval, NAKO: German National Cohort, MZ: German Census Survey

Supplementary Table 2: Comparison of the intersectional population strata between the NAKO and the MZ, stratified by sex and age group.

Intersectional population strata			Female				Male			
			NAKO		MZ		NAKO		MZ	
			Prop. %	95% CI	Prop. %	95% CI	Prop. %	95% CI	Prop. %	95% CI
			**20-29 years**							
**education**	**cohabitation**	**country of birth**								
high	with partner	Germany	29.8	29.0 - 30.7	12.8	12.4 - 13.3	24.4	23.5 - 25.3	7.2	6.8 - 7.5
high	with partner	abroad	3.4	3.0 - 3.7	3.5	3.2 - 3.7	2.3	2.0 - 2.6	1.9	1.7 - 2.1
high	w/o partner	Germany	41.9	40.9 - 42.9	32.1	31.5 - 32.8	44.9	43.9 - 45.8	31.7	31.1 - 32.5
high	w/o partner	abroad	3.5	3.2 - 3.9	4.2	3.9 - 4.5	4.1	3.7 - 4.5	5.0	4.6 - 5.4
medium	with partner	Germany	9.7	9.2 - 10.3	10.7	10.3 - 11.2	7.4	6.9 - 7.9	6.4	6.1 - 6.7
medium	with partner	abroad	0.5	0.4 - 0.7	2.1	2.0 - 2.3	0.5	0.4 - 0.7	1.1	0.9 - 1.2
medium	w/o partner	Germany	6.8	6.4 - 7.3	15.3	14.8 - 15.8	9.8	9.2 - 10.4	19.6	19.1 - 20.1
medium	w/o partner	abroad	0.5	0.4 - 0.6	1.6	1.5 - 1.8	0.7	0.5 - 0.9	1.9	1.8 - 2.1
low	with partner	Germany	1.6	1.4 - 1.9	4.9	4.6 - 5.2	1.7	1.4 - 1.9	4.6	4.3 - 4.8
low	with partner	abroad	0.5	0.4 - 0.6	2.8	2.6 - 3.0	0.5	0.3 - 0.6	1.7	1.6 - 1.9
low	w/o partner	Germany	1.4	1.2 - 1.6	8.2	7.9 - 8.6	3.1	2.7 - 3.4	15.6	15.1 - 16.1
low	w/o partner	abroad	0.3	0.2 - 0.4	1.6	1.4 - 1.8	0.7	0.6 - 0.9	3.3	3.0 - 3.6
			**30-39 years**							
**education**	**cohabitation**	**country of birth**								
high	with partner	Germany	41.2	40.3 - 42.1	22.8	22.3 - 23.3	39.8	38.9 - 40.7	19.4	18.9 - 19.9
high	with partner	abroad	7.2	6.8 - 7.7	7.3	6.9 - 7.6	6.6	6.1 - 7.1	5.1	4.9 - 5.4
high	w/o partner	Germany	17.9	17.2 - 18.6	12.0	11.6 - 12.5	16.8	16.1 - 17.6	13.9	13.4 - 14.4
high	w/o partner	abroad	2.7	2.4 - 3.0	2.6	2.4 - 2.9	2.8	2.5 - 3.1	3.0	2.8 - 3.3
medium	with partner	Germany	15.9	15.2 - 16.6	19.4	18.9 - 19.9	15.7	15.0 - 16.4	14.4	13.9 - 14.8
medium	with partner	abroad	2.0	1.8 - 2.3	4.6	4.4 - 4.9	1.8	1.6 - 2.1	3.5	3.3 - 3.7
medium	w/o partner	Germany	6.0	5.6 - 6.5	8.7	8.3 - 9.0	6.2	5.7 - 6.6	10.8	10.5 - 11.2
medium	w/o partner	abroad	0.7	0.6 - 0.9	1.3	1.2 - 1.5	0.5	0.4 - 0.6	1.3	1.2 - 1.5
low	with partner	Germany	3.0	2.7 - 3.4	8.1	7.8 - 8.5	4.7	4.3 - 5.1	10.1	9.8 - 10.5
low	with partner	abroad	1.4	1.2 - 1.6	6.7	6.4 - 7.0	1.9	1.7 - 2.2	6.1	5.8 - 6.5
low	w/o partner	Germany	1.4	1.2 - 1.7	4.8	4.5 - 5.1	2.7	2.4 - 3.0	9.5	9.1 - 9.9
low	w/o partner	abroad	0.5	0.4 - 0.7	1.7	1.5 - 1.8	0.6	0.4 - 0.7	2.8	2.5 - 3.0
			**40-49 years**							
**education**	**cohabitation**	**country of birth**								
high	with partner	Germany	33.2	32.6 - 33.7	18.4	17.9 - 18.8	38.5	37.9 - 39.1	20.6	20.1 - 21.1
high	with partner	abroad	6.2	5.9 - 6.5	4.4	4.2 - 4.7	5.9	5.6 - 6.2	3.6	3.4 - 3.9
high	w/o partner	Germany	12.8	12.4 - 13.2	7.9	7.6 - 8.2	10.9	10.5 - 11.3	8.5	8.2 - 8.8
high	w/o partner	abroad	2.1	1.9 - 2.3	1.6	1.4 - 1.7	1.5	1.4 - 1.7	1.3	1.1 - 1.5
medium	with partner	Germany	24.3	23.8 - 24.9	26.5	26.0 - 27.0	20.3	19.8 - 20.8	19.2	18.8 - 19.7
medium	with partner	abroad	2.7	2.5 - 2.9	3.7	3.5 - 3.9	2.5	2.3 - 2.7	3.4	3.2 - 3.6
medium	w/o partner	Germany	8.8	8.4 - 9.1	10.2	9.9 - 10.5	6.8	6.5 - 7.1	9.3	9.0 - 9.6
medium	w/o partner	abroad	0.9	0.8 - 1.0	1.1	0.8 - 1.2	0.5	0.5 - 0.6	0.9	0.8 - 1.0
low	with partner	Germany	4.7	4.5 - 5.0	12.1	11.8 - 12.5	6.7	6.4 - 7.1	14.5	14.1 - 14.9
low	with partner	abroad	2.0	1.9 - 2.2	6.3	6.1 - 6.6	2.7	2.5 - 2.9	6.7	6.4 - 7.0
low	w/o partner	Germany	1.6	1.5 - 1.8	5.9	5.6 - 6.2	2.9	2.7 - 3.1	9.7	9.4 - 10.1
Low	w/o partner	abroad	0.7	0.6 - 0.8	1.8	1.7 - 2.0	0.7	0.6 - 0.8	2.2	2.0 - 2.4

Supplemental table 2: (continued)

Intersectional population strata			Female				Male			
			NAKO		MZ		NAKO		MZ	
			Prop. %	95% CI	Prop. %	95% CI	Prop. %	95% CI	Prop. %	95% CI
						50-59 years				
**education**	**cohabitation**	**country of birth**								
high	with partner	Germany	26.9	26.4 - 27.4	14.2	13.9 - 14.6	33.0	32.5 - 33.6	18.7	18.3 - 19.2
high	with partner	abroad	3.8	3.6 - 4.0	3.1	2.9 - 3.3	4.0	3.7 - 4.2	3.1	2.9 - 3.3
high	w/o partner	Germany	12.7	12.3 - 13.1	6.8	6.5 - 7.0	10.0	9.6 - 10.3	6.8	6.5 - 7.0
high	w/o partner	abroad	1.8	1.7 - 2.0	1.3	1.2 - 1.5	1.2	1.1 - 1.4	1.0	0.9 - 1.1
medium	with partner	Germany	27.3	26.7 - 27.8	26.3	25.8 - 26.8	23.5	23.0 - 24.0	19.3	18.9 - 19.8
medium	with partner	abroad	1.9	1.7 - 2.0	2.6	2.4 - 2.7	1.8	1.6 - 2.0	2.7	2.5 - 2.9
medium	w/o partner	Germany	10.8	10.5 - 11.2	9.5	9.2 - 9.8	7.2	6.9 - 7.6	7.2	6.9 - 7.5
medium	w/o partner	abroad	0.8	0.7 - 1.0	1.0	0.9 - 1.1	0.4	0.3 - 0.5	0.7	0.6 - 0.8
low	with partner	Germany	8.3	8.0 - 8.6	20.1	19.6 - 20.5	11.5	11.2 - 11.9	21.7	21.2 - 22.2
low	with partner	abroad	1.8	1.6 - 1.9	5.4	5.1 - 5.6	2.7	2.5 - 2.9	5.8	5.6 - 6.1
low	w/o partner	Germany	3.2	3.0 - 3.4	8.1	7.8 - 8.4	4.0	3.7 - 4.2	11.1	10.8 - 11.5
low	w/o partner	abroad	0.7	0.6 - 0.8	1.7	1.5 - 1.8	0.7	0.6 - 0.8	1.9	1.7 - 2.0
						60-69 years				
**education**	**cohabitation**	**country of birth**								
high	with partner	Germany	20.0	19.6 - 20.5	8.9	8.5 - 9.2	33.3	32.8 - 33.9	18.7	18.2 - 19.2
high	with partner	abroad	3.0	2.8 - 3.2	2.2	2.0 - 2.4	3.4	3.1 - 3.6	2.7	2.5 - 3.0
high	w/o partner	Germany	10.4	10.1 - 10.8	4.5	4.3 - 4.8	7.5	7.2 - 7.8	4.7	4.4 - 4.9
high	w/o partner	abroad	1.8	1.6 - 1.9	1.1	1.0 - 1.3	0.9	0.8 - 1.1	1.0	0.8 - 1.1
medium	with partner	Germany	24.1	23.6 - 24.6	18.6	18.1 - 19.1	21.0	20.5 - 21.5	15.9	15.5 - 16.4
medium	with partner	abroad	1.5	1.3 - 1.6	1.7	1.6 - 1.9	1.5	1.3 - 1.6	1.9	1.8 - 2.1
medium	w/o partner	Germany	10.5	10.2 - 10.9	7.4	7.1 - 7.7	4.2	4.0 - 4.5	3.9	3.7 - 4.2
medium	w/o partner	abroad	0.9	0.8 - 1.0	0.9	0.8 - 1.0	0.4	0.3 - 0.5	0.3	0.3 - 0.4
low	with partner	Germany	17.4	16.9 - 17.9	32.3	31.7 - 32.8	20.0	19.6 - 20.6	31.9	31.3 - 32.5
low	with partner	abroad	1.7	1.5 - 1.9	6.0	5.7 - 6.3	2.4	2.2 - 2.6	7.0	6.7 - 7.4
low	w/o partner	Germany	7.7	7.4 - 8.1	13.6	13.2 - 14.0	4.7	4.4 - 4.9	10.3	9.9 - 10.7
low	w/o partner	abroad	1.0	0.9 - 1.1	2.8	2.6 - 3.0	0.6	0.5 - 0.7	1.6	1.4 - 1.8

Prop.: proportion, CI: confidence interval, NAKO: German National Cohort, MZ: German Census Survey
